# Design and Manipulation of Ferroic Domains in Complex Oxide Heterostructures

**DOI:** 10.3390/ma12193108

**Published:** 2019-09-24

**Authors:** Nives Strkalj, Elzbieta Gradauskaite, Johanna Nordlander, Morgan Trassin

**Affiliations:** Department of Materials, ETH Zurich, Vladimir-Prelog-Weg 4, 8093 Zurich, Switzerland

**Keywords:** multiferroic, magnetoelectric, ferroelectrics, domain inversion, ferroelectric vortices, domain imprint

## Abstract

The current burst of device concepts based on nanoscale domain-control in magnetically and electrically ordered systems motivates us to review the recent development in the design of domain engineered oxide heterostructures. The improved ability to design and control advanced ferroic domain architectures came hand in hand with major advances in investigation capacity of nanoscale ferroic states. The new avenues offered by prototypical multiferroic materials, in which electric and magnetic orders coexist, are expanding beyond the canonical low-energy-consuming electrical control of a net magnetization. Domain pattern inversion, for instance, holds promises of increased functionalities. In this review, we first describe the recent development in the creation of controlled ferroelectric and multiferroic domain architectures in thin films and multilayers. We then present techniques for probing the domain state with a particular focus on non-invasive tools allowing the determination of buried ferroic states. Finally, we discuss the switching events and their domain analysis, providing critical insight into the evolution of device concepts involving multiferroic thin films and heterostructures.

## 1. Introduction

Multiferroic materials with coexisting electric and magnetic order are technologically attractive [1,2,3,4]. In ferroic and multiferroic materials, an area with uniformly oriented order parameter is defined as a domain. A domain wall separates two adjacent domains with different order parameter orientations. In the absence of coupling between the order parameters, the independent access to either the electric or magnetic domain states suggests higher storage density for memory architectures [5,6]. When ferroic states are coupled, correlations of domain pattern are expected and the magnetoelectric coupling can, for instance, enable low-energy-consuming electric-field control of the magnetic order, which is of great interest for ultra-efficient spintronic applications [7,8].

Domain state and domain wall engineering are essential for applications of ferroic thin films since the switching mechanism is driven by nucleation and motion of domain walls. In thin films hosting a reversible electric polarization, the interplay between epitaxial strain and electrostatics renders the control of the domain architecture possible using the lattice and charge degrees of freedom [9]. Optimal device operation puts rigorous requirements on the domain states. Ferroelectric tunnel junctions, in which the tunneling current depends on the polarization state, require the stabilization of the single-domain state for maximized resistance difference [10,11]. Other device paradigms benefit from domain formation, which increases the dielectric response [12,13,14,15], enhances tunneling across tunnel junctions [16] or allows domain pattern inversion [17]. Furthermore, domain walls can have functionalities different from the bulk of domains [18,19] such as enhanced ferroelectricity [20], magnetism [21], multiferroicity [22] or magnetoelectric coupling [23], increased conductivity [24,25], and photovoltaic efficiency [26].

The increased control of complex domain architecture using charged surface states at oxide interfaces, defects in stoichiometry or domain imprint across interfaces, as described in this review, leads to new developments for buried domain states in oxide heterostructures. Here, we place emphasis on the recent advances in domain design and manipulation in ferroelectric and multiferroic thin films and heterostructures. Therefore, the first part of this review is devoted to ferroic domain design in thin layers and superlattices. In the following sections, we review the progress and challenges in accessing the domain state and the switching events in multiferroics, especially in buried layers.

## 2. Controlled Ferroelectric and Multiferroic Domain Architectures in Thin Films

### 2.1. Domain State and Domain Wall Engineering in Ferroelectric Thin Films

The domain state in ferroelectric thin films is set by a combination of factors such as strain, chemistry and charge screening at the interfaces, and layer thickness. Domain engineering in ferroelectric thin films using epitaxial strain has been reviewed in great detail, see [1,27,28] for references. Here, we shed light on recent development in ferroelectric domain engineering using electrostatic boundary conditions and chemistry at oxide interfaces [29,30,31,32,33]. 

The onset of net ferroelectric polarization triggers the accumulation of bound surface charges in ferroelectric materials. This charge accumulation, in return, creates a depolarizing field oriented oppositely to the ferroelectric polarization. The depolarizing field plays a decisive role in determining the domain state and can, for instance, induce domain splitting [34,35] or, in extreme cases, suppress the net ferroelectric polarization [18,36,37].

By acting on the screening of ferroelectric bound charges, the depolarizing field strength can be modulated. The electrostatic environment can be engineered by interfacing ferroelectric layers with metallic/dielectric layers or by changing the environment, i.e., adsorbates and surrounding gas atmosphere. This represents alternative routes towards control of polarization state and domain formation in ferroelectric thin films.

**Figure 1:**
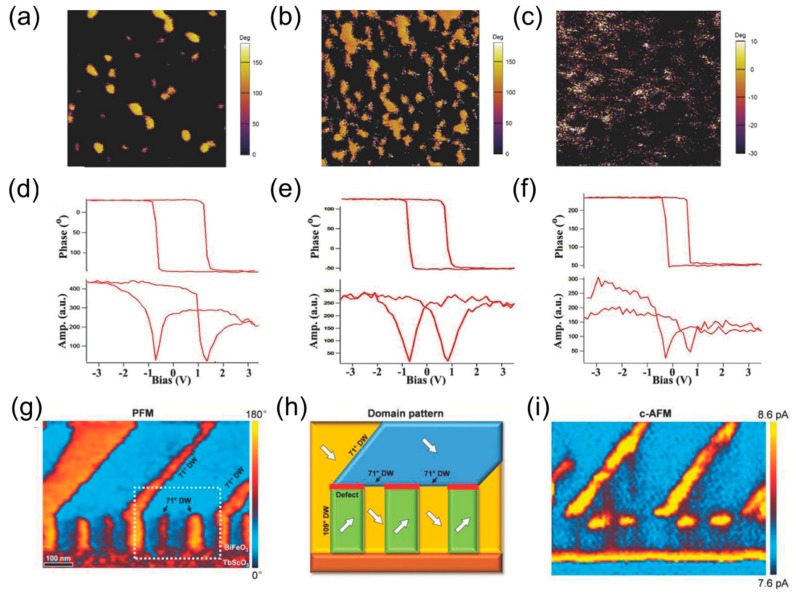
Resonance tracking-piezoresponse force microscopy phase images and switching spectroscopy piezoresponse force microscopy (SSPFM) loops compare the domain state and switching behavior without STO (**a**,**d**), with 3 uc (**b**,**e**) and with 10 uc (**c**,**f**) thick STO spacer in the PZT/STO/PZT heterostructure. Reprinted with permission from [38], copyright Wiley-VCH Verlag GmbH and Co. KGaA, 2015. (**g**–**i**) Cross-sectional PFM phase image (**g**), a sketch of the domain state (**h**) and c-AFM image showing a change of domain pattern from 109° to 71° domain wall type by adding defects during the synthesis of BFO on TSO (**i**). Reprinted with permission from [47], copyright Wiley-VCH Verlag GmbH and Co. KGaA, 2015.

In the case of the prototypical ferroelectric system with uniaxial out-of-plane polarization such as ultrathin Pb(Zr_0.2_Ti_0.8_)O_3_ (PZT), charges accumulate mainly at top and bottom interfaces. The introduction of a metallic buffer provides sufficient charge screening to stabilize a single-domain state. However, intentionally introducing a large depolarizing field can benefit the ferroelectric properties as demonstrated by Liu et al. [38]. Inserting a dielectric SrTiO_3_ (STO) layer in a PZT/STO/PZT heterostructure resulted in faster nucleation speed attributed to preexisting domains induced by depolarizing field in the remanent state and reduced leakage current. Here, the ultrathin STO layer gets partially polarized by the surrounding ferroelectric matrix, even in the presence of domains. Resonance tracking-piezoresponse force microscopy phase images and switching spectroscopy piezoresponse force microscopy (SSPFM) loops in Figure 1a–f compare the domain state and switching behavior in heterostructures without STO, with three unit cells (uc) and 10 uc thick STO layers. Inserting a 3 uc thick STO layer results in domain formation and a drastic decrease of built-in voltage (shift of the ferroelectric hysteresis towards negative/positive voltages). When the thickness of the dielectric is further increased to 10 uc, the effect vanishes as the heterostructure starts behaving as two decoupled PZT layers. 

In BiFeO_3_ (BFO) thin films, a monodomain state [39], periodically striped domains [40,41,42] or arrays of flux-closure domain pattern [43] can be achieved by modifying the electrostatic boundary conditions. Monodomain states and configurations with 71° domain walls experience large depolarizing fields due to a single out-of-plane polarization component and can only be stabilized on conducting buffer layers providing sufficient charge screening. In the absence of a metallic buffer, the BFO polarization state tends to evolve into a configuration with 109° domain walls for which the out-of-plane component and the corresponding surface charges cancel out [42,44]. Ferroelectric flux-closure domains self-assemble near the interface of the BFO film and an insulating TbScO_3_ (TSO) substrate. Another path opening the range of control over the domain state in ferroic thin films consists of introducing defects in stoichiometry during the growth process [45,46]. Recently, Li et al. revealed that defects in stoichiometry can influence the domain architecture of thin films [47]. Temporarily changing the growth temperature during the deposition using oxide molecular beam epitaxy of multiferroic BFO thin film on TSO substrate results in an Fe_2_O_3_ defect layer which affects the domain structure. At the negatively charged Fe_2_O_3_ defect layer, the initial 109° domain wall configuration changes to a 71° domain wall configuration. Figure 1g–h shows the resulting change of domain pattern and the induced formation of charged domain wall, confirmed by cross-sectional conducting atomic force microscopy (C-AFM), see Figure 1i.

### 2.2. Engineering Multiferroic Domains in Thin Films

In a multiferroic system with coexisting ferroelectric and (anti-)ferromagnetic orders [48], domains can be defined for each order parameter and they may or may not be coupled. Hence, the resulting domain architecture gains in complexity but also in functionality. Materials with coupled ferroic domains, i.e., multiferroic domains, include type I multiferroics (e.g., BFO [49], YMnO_3_ [50,51]) where the order parameters establish independently, exhibiting different ordering temperatures, and type II multiferroics where both ferroic order share a common origin (e.g., orthorhombic TbMnO_3_ [52,53], TbMn_2_O_5_ [54], MnWO_4_ [55]). For reviews on the general topic of multiferroic and magnetoelectric materials, see, for example, [3,56,57].

**Figure 2:**
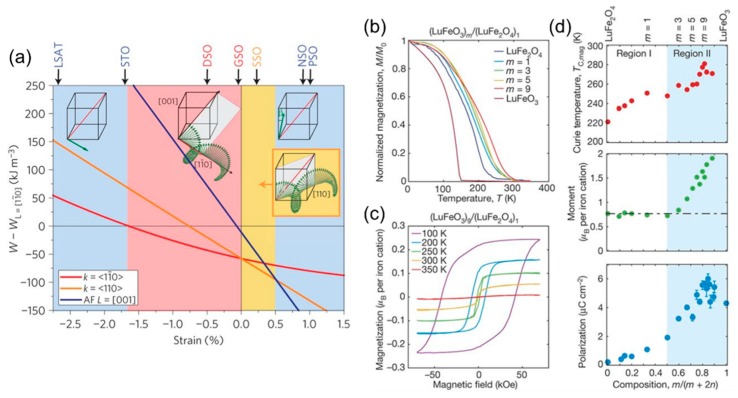
(**a**) The energy of three magnetic states in BFO thin films (cycloid with propagation direction along <$1\bar{1}0$>, along <110>, and collinear antiferromagnetic order close to [001]) relative to the energy of collinear antiferromagnetic order with antiferromagnetic vector along in-plane [$1\bar{1}0$]. Tuning the in-plane strain results in stability of different states. Reprinted with permission from [60], copyright Springer Nature, 2013. (**b**,**c**) Magnetization vs. temperature for a series of (LuFeO_3_)_m_/(LuFe_2_O_4_)_1_ superlattices and the magnetization loops as a function of the magnetic field for the (LuFeO_3_)_9_/(LuFe_2_O_4_)_1_ superlattice. (**d**) The ferromagnetic Curie temperature, the total moment per iron cation in LuFe_2_O_4_ at 50 K assuming the moment of LuFeO_3_ remains constant and average polarization from high-angle annular dark-field imaging in scanning transmission electron microscopy for superlattice layering plotted as a function of composition. Regions I and II show data for the (LuFeO_3_)_1_/(LuFe_2_O_4_)_n_ and (LuFeO_3_)_m_/(LuFe_2_O_4_)_1_ series, respectively. Reprinted with permission from [63], copyright Springer Nature, 2016.

In thin-film heterostructures, the study of multiferroic domains is largely focused on BFO-based systems. The seminal work from Wang at al. [49] demonstrated the coexistence of ferroelectric and weak ferromagnetic state in compressively strained epitaxial thin films at room temperature. In domain-engineered BFO-based heterostructures, this led to the full net magnetization reversal via magnetoelectric coupling with either in-plane or out-of-plane electric field [58,59]. Sando et al. [60] further observed a control of the spin cycloid propagation direction in the antiferromagnetic state by various epitaxial strain states (Figure 2a), promising for magnonics [61] and spintronics with antiferromagnets [62]. With the progress in thin film epitaxial design, new multiferroic materials have been realized, beyond the realm of the BFO prototypical perovskite structure by nanoscale engineering of additional order parameters through strain and epitaxy [63]. Such systems are to be distinguished from so-called composite or artificial multiferroics where coupling typically appears at the interfaces between two separate ferroic materials guiding their individual domain formation (see section “Domain pattern transfer in artificial multiferroic heterostructures”). 

In ferroelectric-LuFeO_3_/ferrimagnetic-LuFe_2_O_4_ superlattices, the coveted coexistence and coupling of ferroelectric and magnetic order at room-temperature were achieved by epitaxial stabilization [63]. Multiferroicity could be demonstrated by propagating the improper geometrically driven polarization order parameter from LuFeO_3_ into the ferrimagnetic LuFe_2_O_4_ in short-period superlattices. In atomically engineered multilayers, the cooperative interplay of the ferroelectric and ferrimagnetic order was shown and an increased magnetic transition temperature in LuFe_2_O_4_ was measured once inserted into the ferroelectric-LuFeO_3_/ferrimagnetic-LuFe_2_O_4_ superlattice, see Figure 2b–d.

### 2.3. Domain Pattern Transfer in Artificial Multiferroic Heterostructures

The lack of single-phase magnetoelectric multiferroics, exhibiting simultaneously strong and coupled magnetic and ferroelectric orders at technologically relevant temperatures, is driving the increased effort into the development of so-called artificial multiferroic heterostructures. In artificial multiferroic heterostructures, these orders are combined by assembling different ferroic materials [1,64,65]. This can be achieved by designing ferromagnetic columnar nanostructures in a ferroelectric matrix [66,67,68,69] or in bilayer heterostructures. We will here focus on the latter case, where domain investigations have been reported. 

The interface between ferroelectric and ferromagnetic thin films is particularly compelling-aside from ordinary interface effects, like those arising from strain and broken inversion symmetry, interfacing two ferroic orders can enable magnetoelectric coupling and the imprint of ferroelectric domain architecture into the adjacent ferromagnetic domain state. In artificial multiferroic heterostructures, the application of an electric field can result in a net change in the magnetic order of the ferromagnetic component. There are three distinct ways to achieve magnetoelectric coupling [64]: Via (i) strain, (ii) direct (spin) exchange and (iii) charge co-upling. The electrical control of magnetism has been reviewed in great detail previously, here we restrict ourselves to the visualization of domain imprint across the ferroelectric/ferromagnetic interface.

**Figure 3:**
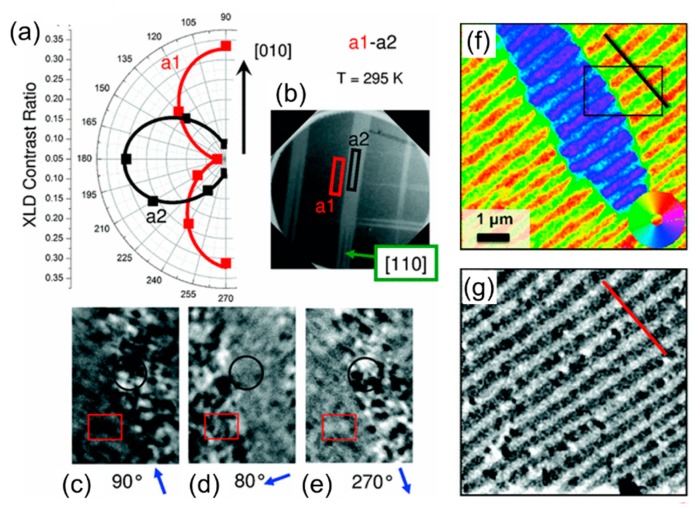
(**a**) Angular dependence of linear dichroism in CFO at Fe L_2_ edge for a1-a2 type BTO domains at 295 K, with XLD domains in (**b**) measured at *θ* = 180◦ and arrow indicating the in-plane [110] substrate direction. (**c**–**e**) X-ray magnetic circular dichroism (XMCD) domains in 7 μm × 11 μm area for a1 (stripe with red box) or a2 (stripe with a black circle) domain variants for various angles *θ* with the x-ray incidence direction shown with small arrows, showing uniaxial in-plane magnetic anisotropy. Adapted from reference. Reprinted with permission from [71], copyright the American Physical Society, 2012. (**f**) Scanning electron microscopy with polarization analysis (SEMPA) image of CoFe magnetization (direction indicated by the color wheel) and (**g**) simultaneously acquired BSE image of underlying ferroelectric domain structure. Reprinted with permission from [41], copyright the American Physical Society, 2013.

(i) In strain-coupled artificial multiferroics, a piezoelectric and a magnetostrictive layer are elastically coupled once the magnetoelastic anisotropy in the system becomes stronger than the magnetocrystalline anisotropy [70]. This leads to controllable magnetoelastic anisotropy due to propagation of epitaxial strain across the interface. One instructive example investigated by Chopdekar et al. is CoFe_2_O_4_ (CFO)–BaTiO_3_ (BTO)-- composite [71]. The strain-induced variation in the ferromagnetic state was imaged via x-ray linear dichroism (XLD) (Figure 3a,b) and related to corresponding in-plane and out-of-plane BTO ferroelectric domains. X-ray magnetic circular dichroism (XMCD) was used to spatially resolve the domain pattern in ferromagnetic CFO (Figure 3c–e) imprinted by ferroelectric BTO-- domains. This experiment shows that different in-plane (a1, a2) and out-of-plane (c) ferroelectric domains have a one-to-one correlation to magnetic domains with varying magnetic uniaxial anisotropy. Similar effects were observed in other ferromagnets: CoFe [72], CoFeB [73,74], Fe [75], La_1-x_Sr_x_MnO--_3_ (LSMO) [76], Ni [77], and NiFe [78]. A key requirement for these observations is strong elastic pinning of magnetic domain walls onto ferroelectric domain walls [79].

(ii) In exchange-coupled multiferroic composites, the interaction occurs between a ferromagnet and a single-phase multiferroic magnetoelectric with uncompensated antiferromagnetic order. The electric field acts on both ferroelectric polarization and the direction of antiferromagnetic spin ordering in the multiferroic. This allows a direct exchange effect between the antiferromagnetic and ferromagnetic spin orderings. Although the physics of exchange coupling is different from that of strain coupling, both produce lateral modulations of magnetic anisotropy leading to domain transfer. Trassin et al. [41] determined the interfacial coupling with spatial resolution in the prototypical multiferroic Co_90_Fe_10_ (CoFe)/BFO heterostructure by imaging magnetization using scanning electron microscopy with polarization analysis (SEMPA) and underlying polarization with back-scattered electrons (BSE) (Figure 3f,g). A one-to-one coupling between the BFO ferroelectric domain and the weak ferromagnetic moment results in a domain transfer into the adjacent exchanged-coupled ferromagnetic layer CoFe grown on top of the BFO layer. Induced uniaxial magnetic anisotropy is rationalized by an interfacial exchange coupling between the CoFe moments and the canted antiferromagnetic moment in BFO. Similar exchange coupling was observed for other ferromagnets, such as LSMO [80], NiFe [81], Co [82]. Although BFO is undoubtedly the most promising multiferroic, operational at room temperature, other single-phase multiferroics like YMnO_3_ [83] and LuMnO_3_ [84] can also induce exchange coupling. 

(iii) Charge-coupled artificial multiferroics make use of the ferroelectric field effect: Bound charges at the ferroelectric interface are screened by the ferromagnetic layer, leading to either accumulation (hole-doped) or depletion (electron-doped) states in the ferromagnet when the ferroelectric polarization is pointing away from or towards the ferromagnet, respectively. If the ferromagnet is a strongly correlated system, this can result in drastic alterations of magnetization and even domain imprint. This mechanism is different from the two mentioned previously, because changes in magnetization are limited to interfaces, up to a screening length [85]. However, full domain transfer could be achieved in the ultrathin regime. The effect has been most widely studied in LSMO [86,87,88] interfaced with ferroelectrics, such as BTO and PZT. It has also been predicted for ferromagnetic metals [89,90] and SrRuO_3_ (SRO) [91].

## 3. Accessing the Domain State in Ferroic Multilayers

Domain investigation is a critical element in the understanding of complex oxide multifunctional layers [92] since the order-parameter coupling, and interfacial properties express themselves in the ferroic domain architecture. In thin films, the reduced domain dimension to the nanoscale adds to the difficulty of independently probing multiple ferroic states coexisting in a single phase. Despite tremendous advances in understanding ferroelectrics, fundamental aspects of their behavior once inserted in multilayers or superlattices remain unclear. The main reason is the intricate nature of the interactions between polar and non-polar layers and the difficulty to access buried ferroelectric states in heterostructures.

**Figure 4:**
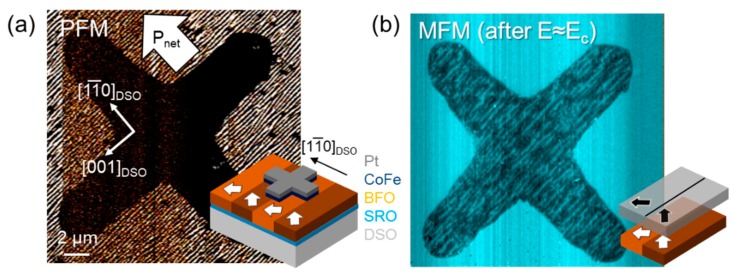
(**a**) Magnetoelectric characterization of the Pt/CoFe/BFO/SRO//DSO heterostructure. The in-plane PFM response reveals an FE stripe-domain distribution in BFO, yielding an in-plane net polarization P_net_ (white arrow). The cross-shaped metallic electrode prevents the observation of the buried FE BFO domain structure. In the inset, the multilayer structure is depicted. (**b**) MFM performed after application of an electric poling field close to the BFO coercive field E_c_ yields magnetic stripes with the same orientation as in the BFO film not covered by CoFe. Reprinted with permission from [93], copyright the American Physical Society, 2018.

By design, the ferroelectric component in artificial multiferroic heterostructures is covered by a conducting ferromagnetic layer. Non-crystalline, oxidation-sensitive ferromagnetic layers need to be grown on top of a ferroelectric layer, after the high-temperature ferroelectric material deposition process. In the case of crystalline ferromagnetic layers for strain-induced interfacial coupling, the ferroelectric film plays the role of the substrate to influence the ferromagnetic lattice and therefore lies underneath the metallic layer. This buried nature results in a loss of information about the ferroelectric domain architecture when using conventional techniques such as PFM. The magnetic domain state of the top layer can, however, be accessed directly with scanning probe microscopy, see Figure 4a,b [93,94] or techniques such as magneto-optical Kerr effect (MOKE) or photoemission electron microscopy (PEEM) [95,96].

### 3.1. Probing Buried Polarization States (Invasive)

#### 3.1.1. Scanning Transmission Electron Microscopy (STEM)

**Figure 5:**
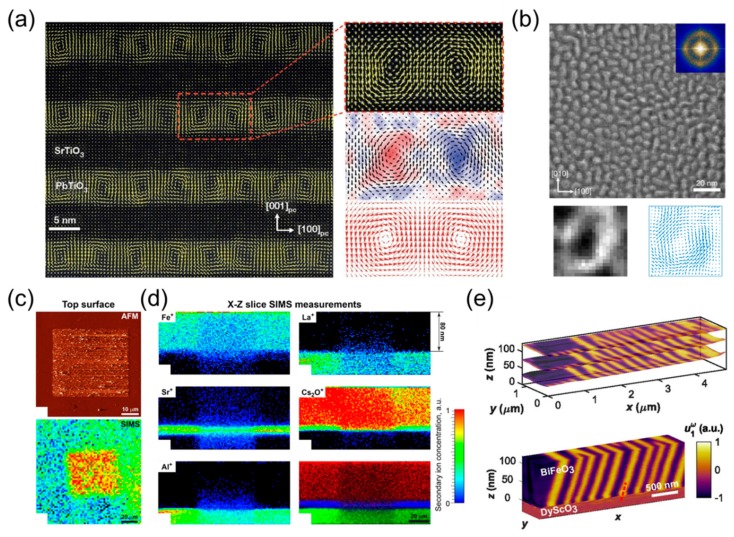
(**a**) Cross-sectional high-resolution STEM image with an overlay of the polar displacement vectors for a PTO_10_/STO_10_ superlattice on DyScO_3_ (DSO). A magnified image of a single vortex–antivortex pair, the curl of the polar displacement and the polarization vectors from a phase-field simulation of the same superlattice. Reprinted with permission from [100], copyright Springer Nature, 2016. (**b**) Planar-view dark-field STEM imaging shows the widespread occurrence of circular features in a (PTO_16_/STO_16_)_8_ superlattice with an inset of the FFT of the image, corresponding to the reciprocal space studies. Below are the images of a single skyrmion bubble with the mapped polarization vectors. Reprinted with permission from [101], copyright Springer Nature, 2019. (**c**) Top surface topography and secondary Bi^+^ ions distribution. (**d**) XZ depth profiles of secondary ion concentrations. Reprinted with permission from [104], copyright 2016 American Chemical Society. (**e**) Tomographic reconstruction of ferroelectricity from a BFO thin-film heterostructure. Reprinted with permission from [107], copyright 2019 National Academy of Sciences.

Advanced microscopy techniques such as STEM are becoming standard for highly resolved polarization mapping within heterostructure cross-sections and in the planar view [97]. The atomic displacements corresponding to the ferroelectric polarization can be mapped out for domains extending along the zone axis directions. This powerful tool allowed the first experimental observation of flux closure domain patterns [98], Néel type ferroelectric domain wall [99], ferroelectric vortices [100] and skyrmions [101] in PbTiO_3_/STO (PTO/STO) superlattices (Figure 5a,b), but remains a destructive analysis. Further progress in accessing the domain dynamics using differential phase contrast [102,103] and displacement mapping of switching at the atomic resolution are anticipated.

#### 3.1.2. Time of Flight Secondary Ion Mass Spectrometry (TOF-SIMS)

Another approach towards the determination of buried polar states consists of probing the surface chemistry of ferroelectric films. Polarization switching events have mostly been analyzed with the assumption of unchanged stoichiometry. Ievlev et al. [104] revealed the chemical state evolution after a ferroelectric switching event in BFO thin films using TOF-SIMS. The SIMS technique sputters off ions from the film and therefore addresses the depth profile of the cation composition. The local application of an electric field is accompanied by a redistribution of the base cation (Bi^+^ and Fe^+^, but also adsorbates). This phenomenon concerns the entire switched volume. The chemical profiles of various elements are mapped after a local switching event in Figure 5c,d. The change in the contrast of cations population correlates with the poled volume within the ferroelectric film.

With the increasing knowledge of the impact of surface chemistry on switching properties of ferroelectric materials, these works [94,105] further suggest surface chemistry as a tool to probe polar states in complex oxide thin films in device designs and in superlattice architectures.

#### 3.1.3. Piezoresponse Force Microscopy (PFM) 

Nanoscale domain patterns in ferroelectric thin films are most commonly imaged by PFM [106]. However, PFM is surface-sensitive and lacks the resolution in-depth, i.e., access to the volume distribution of nanosized domains and domain walls. Towards the investigation of nanoscale domains within the films thickness, Steffes and coworkers [107] have been developing a scanning probe technique using a combination of PFM and nanomaching. In tomographic domain analysis (Figure 5e), the surface material is progressively removed via mechanical friction with the scanning tip, exposing successive sections of the domain state down to the substrate. This technique, however, puts some strict restrictions on the operation conditions since mechanical stress can drastically impact the ferroelectric state in thin layers [108].

### 3.2. Non-Invasive Probe of Buried Ferroelectric Domains

#### 3.2.1. X-Ray Diffraction

X-ray diffraction of thin films is an effective non-invasive probe of ferroelectric polarization via measurement of structural parameters. In single layers, tetragonality and thus indirectly polarization can be accessed through measurements of the out-of-plane and in-plane lattice parameters. However, detection in the ultrathin regime is limited by the reduced sample volume. The superlattice architecture is, therefore, used to reproduce ultrathin film behavior while providing increased active volume. The ferroelectric/dielectric superlattices became the model system for such analysis [109,110]. It revealed the unprecedented capabilities of x-ray diffraction applied to thin films with periodic domain architectures [111,112,113,114,115]. Furthermore, nanofocused x-ray diffraction imaging [116,117,118] is an emerging approach to locally probe domain and domain wall architectures in thin films.

**Figure 6:**
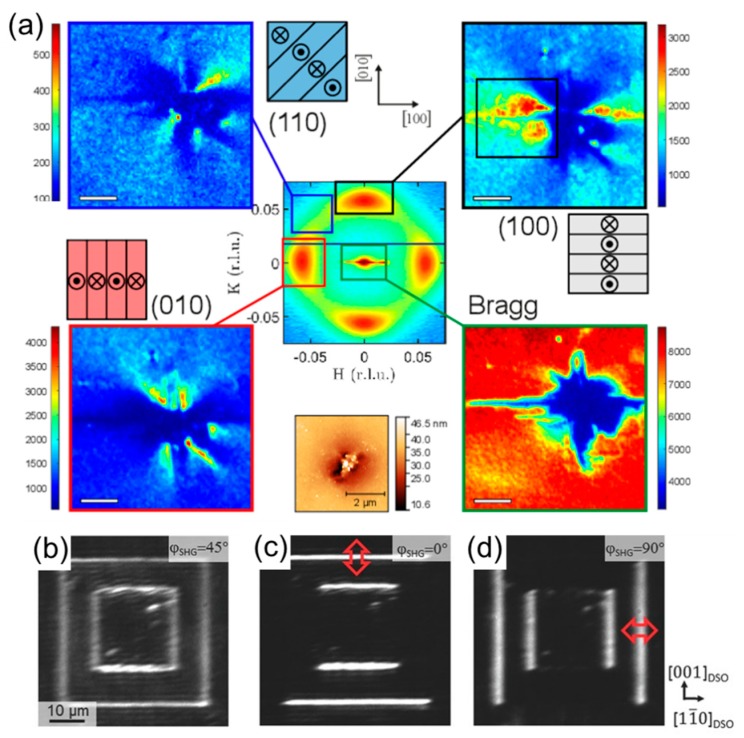
(**a**) In-plane local reciprocal space maps (RSM) around the out-of-plane (002) Bragg reflection with satellite peaks on a diffuse scattering ring corresponding to the stripe domain periodicity in the PTO/STO superlattice. Spatially scanning the area at the x-ray angles of the satellite peaks in the (100), (110) and (010) direction around the defect shows the domain type dependence on the defect shape, i.e., the domains lie parallel to the lines of the defect. Reprinted with permission from [119]. (**b**) SHG images of voltage-poled 180° domain walls in PZT films grown on SRO buffered (DSO) show the total in-plane polarization, the polarization parallel to the (**c**) [001] DSO direction and (**d**) [110] DSO direction. Reprinted with permission from [99], copyright Wiley-VCH Verlag GmbH and Co. KGaA, 2016.

Hadjimichael et al. [119] recently demonstrated the potential of diffuse scattering for nanoscale investigation of domain and domain wall architecture in ferroelectric PTO/STO superlattices. Local reciprocal space maps (RSM) were measured around the out-of-plane (002) Bragg PTO reflection. The additional periodicity emerging from nanoscale domain ordering can be directly addressed by analyzing satellite peaks on the diffuse scattering ring. By selecting the corresponding x-ray angles and scanning the sample area, the domain structure can be spatially mapped out. Figure 6a shows the results demonstrating the impact of a topographical defect on the superlattice domain distribution. This establishes graphoepitaxy [120], i.e., influencing epitaxial growth using substrate patterning, as a route for domain engineering in thin film heterostructures. Resonant x-ray diffraction experiments performed on multiferroic GaFeO_3_ thin films [121] further demonstrated the efficiency of diffraction-based techniques for analysis of cationic distribution [122] and polarization in oxide thin films [123].

#### 3.2.2. Optical Second Harmonic Generation (SHG)

Optical SHG is a non-destructive, non-invasive probing technique for polarization in ultrathin ferroelectric films. The SHG technique is sensitive to inversion symmetry breaking and, therefore. ideal for probing ferroelectricity. Previously devoted to bulk ferroic materials investigation, SHG has become an essential tool for the examination of ferroelectric domains in thin films. Its potential as a ferroic state probe has been the topic of several reviews dealing with bulk and thin film materials [92,124,125].

Despite the lack of spatial resolution (optical resolution limit), a net polarization can be optically detected in domain-engineered films, exhibiting either a single-domain state or a domain architecture leading to a net polarization. The polarization analysis using SHG is not affected by increasing leakage currents in the low thickness range, which prevent the determination of intrinsic ferroelectric behavior using the conventional ferroelectric testing approach. The SHG investigation, therefore, enables the determination at the ultrathin limit of ferroelectricity.

Recently, a specific non-linear optical signature of tilted 180° ferroelectric domain walls corresponding to a mixed Ising–Néel domain-wall type was shown for the first time (Figure 6b–d) [99,126]. This observation, confirmed by STEM imaging, challenges the expectation of Ising-like 180° ferroelectric domain walls in ferroelectric thin films. Furthermore, the exceptional capability of SHG to probe the ferroelectric domain architecture within the volume was demonstrated [127]. This is critical for the understanding of domain wall motion during switching events [128].

### 3.3. Probing Magnetic Domain Architectures (Non-Invasive)

#### 3.3.1. Magnetic Force Microscopy (MFM) 

**Figure 7:**
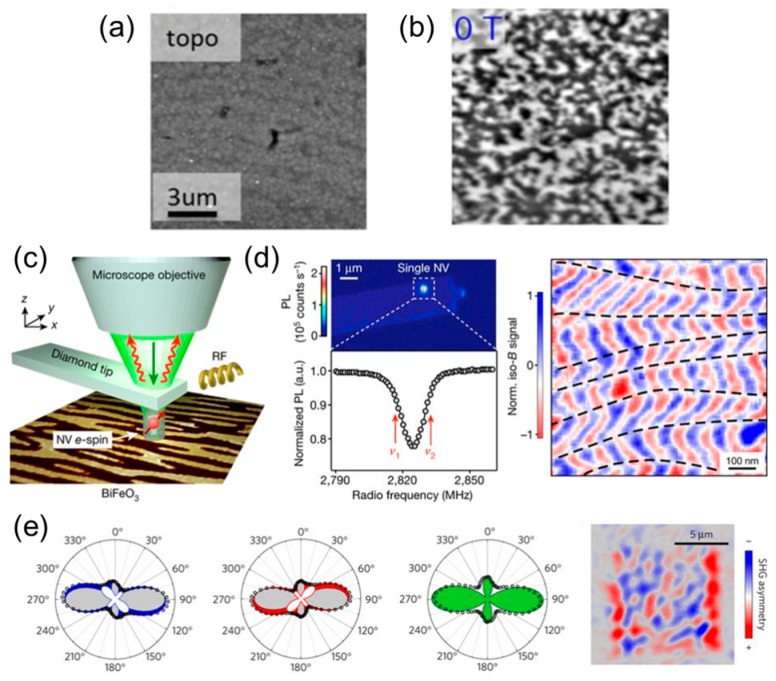
(**a**,**b**) Topographic, and MFM image of the h-LuFeO_3_ film at 50 K after zero-field cooling. Reprinted with permission from [133], copyright the American Physical Society, 2017. (**c**) The electronic spin of a single NV defect placed at the apex of a diamond scanning-probe tip is used as an atom-sized magnetic field sensor. (**d**) Top panel, photoluminescence scan of the diamond scanning-probe showing the bright emission from a single NV defect. Bottom panel, spectrum recorded while applying a bias field along the NV axis. Magnetic field image of the BFO film, the black dashed lines are attributed to ferroelectric domain walls leading to abrupt rotations of the cycloidal propagation vector. Reprinted with permission from [129], copyright Springer Nature, 2017. (**e**) Polar plots of the SHG dependence on the incident light polarization direction (with vertical analysis) with fits of the SHG data, composed of the time-invariant (ferroelectric) part (grey areas) and the time-noninvariant (antiferromagnetic) part (white areas). Reconstructed image of the antiferromagnetic texture in BFO sample, color scale representing the asymmetry of the SHG polar plots. Reprinted with permission from [141], copyright Springer Nature, 2019.

Magnetic domain pattern investigation at the nanoscale is nowadays enabled by the development of magnetic force microscopy (MFM) operating under magnetic fields and cryogenic temperatures. The developments in low-temperature scanning probe microscopy led to major improvements in measurement sensitivity [129,130,131]. In MFM, a nanosized magnetized probe tip responds to magnetic stray fields emerging from magnetic samples. However, most of the multiferroic systems exhibit an antiferromagnetic order, having no net magnetic moment and therefore no stray fields. In some cases, the Dzyaloshinskii–Moriya interaction (DMI) results in a symmetry-allowed spin canting and, therefore, the appearance of a weak ferromagnetic moment which can be picked up by MFM [52,57,132]. In the multiferroic hexagonal rare-earth ferrites (h-RFeO_3_), the weak ferromagnetic behavior has been directly observed using MFM at 50 K, see Figure 7a,b [133]. In this work, the ability to probe small canted moments down to 0.002 µB/fu was demonstrated. 

#### 3.3.2. Single Spin Magnetometry

The single-spin magnetometer technique is currently pushing the minimum detectable magnetic moment to even lower values (a few femtotesla). It is based on a point-like impurity nitrogen-vacancy (NV) defect in diamond [134,135,136,137,138] mounted on a scanning tip, which provides probing with excellent spatial resolution [139]. This scanning-probe technique was used to spatially resolve the local magnetic order in multiferroic antiferromagnetic BFO thin films (Figure 7c,d) [129]. A periodic modulation in the magnetic response corresponding to a spin cycloid was measured, and a correlation with the ferroelectric domain architecture obtained by PFM was demonstrated in the magnetoelectric system.

#### 3.3.3. Optical SHG

In some cases, SHG is sensitive to the reduction of symmetry through the magnetic ordering of spins. In a seminal demonstration of this concept, antiferromagnetic domains were imaged in Cr_2_O_3_ magnetoelectric crystal using optical SHG [140]. More recently, SHG has been used to identify the antiferromagnetic contribution of the SHG dependence on incident light polarization and to probe sub-micron sized antiferromagnetic domains in BFO thin films (Figure 7e) [141]. Furthermore, this optical tool can be used for ultrafast dynamics investigations such as the recent example of tracking motion of antiferromagnetic order parameter in YMnO_3_ crystals [142].

## 4. Switching Events in Multiferroics

As described above, the domain analysis post switching provides critical insight into the ferroic behavior. Efforts are now focusing on operando measurements, i.e., probing evolving magnetic and electric domain states during the application of an external field. The investigation of multiferroic switching dynamics, involving domain wall motion, is expected to lead to discoveries beyond the determination of the switching time-scale. The investigation of artificial multiferroic systems is accompanied by the challenge of observing a buried switching event operando.

### 4.1. Imaging a Multiferroic Magnetoelectric Switch

In ferroic materials, beyond the iconic square-like hysteresis of the macroscopic response subject to an applied conjugate field, an understanding and control of ferroic order at the domain level is highly desired. A magnetoelectric multiferroic switch can express itself as a change of electric (magnetic) domain state under the application of a magnetic (electric) field in the remanent state. Magnetoelectric behavior can be demonstrated at the scale of a single ferroic domain and domain walls. The few existing measurements on dynamics of magnetoelectric switching are based on optical SHG and PFM imaging with SHG having the advantage of operando probing the domain state locally during the magnetoelectric switch. 

**Figure 8:**
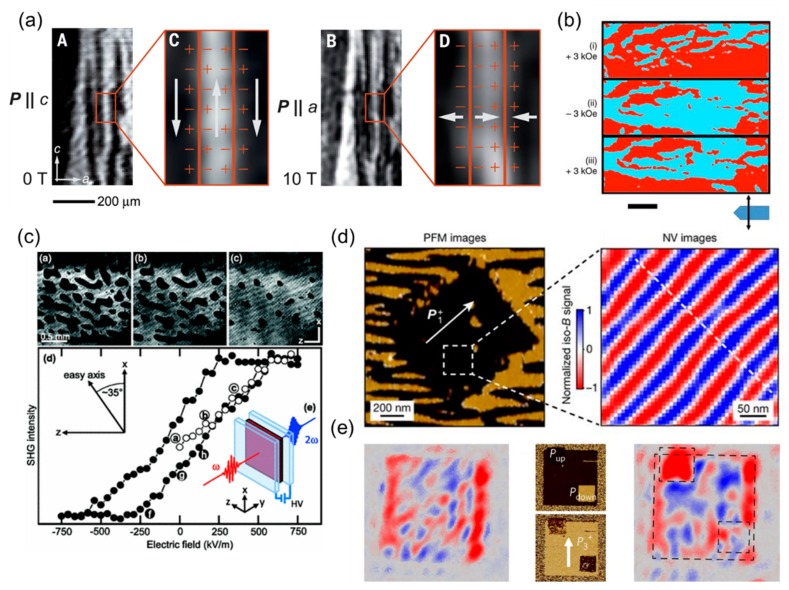
(**a**) SHG images of the polarization flop from Pc to Pa, driven by a reorientation of the spin-cycloidal plane from bc to ab with the application of a magnetic field. Reprinted with permission from [53], copyright AAAS, 2015. (**b**) PFM phase dependency on the orientation of the magnetic field applied to the PZTFT lamella. Growth of regions with polarization direction indicated by red with the application of 3 kOe perpendicular to the lamellar surface, contraction of these regions with an application of -3 kOe. Scale bar, 2  μm. Reprinted with permission from [143]. (**c**) Evolution of the magnetic domain structure in multiferroic MnWO_4_ during quasi-static electric-field poling and the SHG hysteresis loop. Reprinted with permission from [144], copyright the American Physical Society, 2011. (**d**) In-plane PFM image of ferroelectric micrometer-sized domains and the corresponding magnetic field distribution recorded with the scanning-NV magnetometer. (**e**) Antiferromagnetic configurations from SHG image of ferroelectric single domain state (**left**), the PFM images of a local application of an electric field (**middle**) and the SHG image of the antiferromagnetic configuration after the polarization switch (**right**). Reprinted with permission from [129], copyright Springer Nature, 2017.

Figure 8a,b shows examples of the change in the ferroelectric domain state induced by the magnetic field. In the prototypical multiferroic TbMnO_3_, SHG imaging revealed the domain architecture during the polarization flop induced by magnetic field [53]. The spatially resolved information led to the demonstration of the deterministic nature of the phase transition and the formation of charged domain walls in spin-driven ferroelectric multiferroics. 

In a multiferroic solid solution between lead zirconium titanate (PZT) and lead iron tantalate (PFT), PZTFT, PFM measurements showed a change in the ferroelectric domain population under an application of a magnetic field [143].

The desired energy-efficient control of magnetic order by electric field is shown in Figure 8c–e. The pioneering experiment dealt with bulk MnWO_4_ single crystals [144]. The magnetic response induced by electric field was optically probed by SHG through transparent electrodes used for the electric field application (Figure 8c). This direct access to domain state dynamics led to the establishment of the magnetoelectric switching at the millisecond timescale. More recently, a local ferroelectric switch created using PFM in multiferroic BFO films were shown to affect the antiferromagnetic order imaged either by NV center magnetometry or SHG [129,141] (Figure 8d,e). The propagation direction of a spin cycloid or the reset of the domain pattern was demonstrated to depend on the ground state of the system. 

### 4.2. Controlling Domain Dynamics 

**Figure 9:**
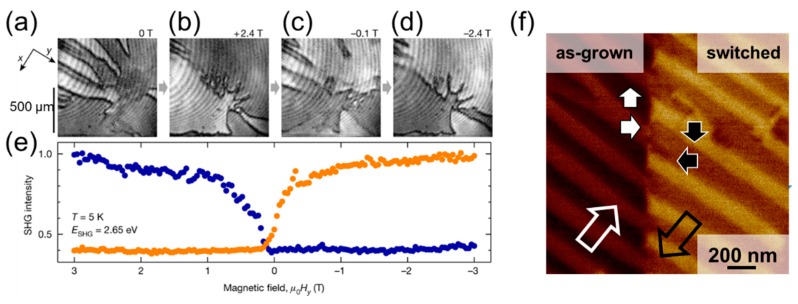
(**a**–**d**) Sequentially taken SHG images of the ± Mx,z domain pattern on a z-oriented Co_3_TeO_6_ at the given magnetic fields H_y_. (**e**), Dependence of the Mx,z domain state on a magnetic field Hy. The domain-state-sensitive SHG interference was measured on two spots of 500 μm diameter that lie in opposite domain states (blue and orange data points). Tuning the magnetic field between positive and negative values reverses the magnetization of each domain while the domain pattern remains intact. Reprinted with permission from [17], copyright Springer Nature, 2018. (**f**) In BFO films grown on DSO, the combination of elastic and electrostatic boundary conditions results in an unchanged domain pattern after local electric field application, before (**left side**) and after (**right side**).

The epitome of controlled domain evolution during switching events is perhaps the inversion of domain pattern, i.e., a switching event which reverses the ferroic order parameter in each domain, but the initial domain pattern is perfectly reproduced. For example, coupling between a complex set of order parameters in multiferroics can allow to independently switch one order parameter with an external field while another retains the memory of the domain pattern. The generality of the concept was demonstrated in the work of Leo et al. [17] by achieving domain inversion in both multiferroic Mn_2_GeO_4_ and magnetoelectric Co_3_TeO_6_, see Figure 9a–e. Other systems exhibiting the inversion of the domain pattern are expected to be discovered. The order-parameter coupling described above can, for instance, be allowed only at the domain walls. The hexagonal manganite family of compounds might be a possible candidate for such domain inversion phenomena. In these materials, the linear magnetoelectric coupling is symmetry-forbidden in the bulk [56,57]. However, SHG experiments revealed that ferroelectric and antiferromagnetic domain walls are coupled [50].

As an alternative to multiple order parameters, the epitaxial strain could play a critical role in the deterministic interexchange of domain patterns. In complex oxide thin films, strain engineering can be used to control the domain pattern. In the case of multiferroic films BFO films grown on DSO, the anisotropic strain state induced by the (110)-oriented orthorhombic substrate results in a stripe-like domain pattern [42]. 

This anisotropic domain architecture imposes periodic electrostatic and elastic boundary conditions at each domain wall which can preserve the memory of the initial domain pattern, and hence the ferroelectric (multiferroic) domain state. The magnetization reversal induced by an electric field in multiferroic BFO thin films relies precisely on such a memory effect of the domain pattern. For a given range of pulse widths and electric field amplitudes, a switching event occurs within each domain. The combination of elastic and electrostatic boundary conditions results in an unchanged domain pattern after local polarization rotation [58] or reversal [59,145]. Figure 9f shows the PFM analysis of a stripe-like BFO domain pattern. The stripe-like domain pattern is preserved after the electric field application, but the polarization is 180° switched in each domain. This ferroelectric switching is accompanied by the corresponding reversal of the local magnetic order in each domain. 

This further suggests strain engineering as a possible route towards the inversion of domain pattern in a wide variety of ferroic systems.

### 4.3. Evolution of Magnetoelectric Coupling in Artificial Multiferroic Heterostructures

**Figure 10:**
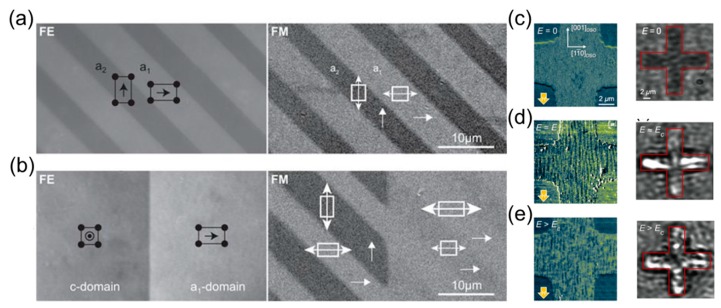
(**a**) Ferroelectric (FE) and ferromagnetic (FM) microstructure after CoFe film growth on BTO and (**b**) the application of an out-of-plane electric field of 10 kV/cm imaged using optical polarization microscopy techniques. Reprinted with permission from [70], (**c–e**) (**right**) The spatially resolved MFM scans show the magnetic domain structure of CoFe after the application of the indicated electric field. MFM scans are recorded in the presence of an in-plane magnetic field of μ_0_H = 50 mT along [001]_DSO_, (**left**) spatially resolved SHG images of buried ferroelectric BFO domains after application of indicated electric fields. The red contour line highlights the area of the cross-shaped CoFe electrode. Reprinted with permission from [93], copyright the American Physical Society, 2018.

Artificial multiferroic heterostructures hold promises for oxide electronics with low-power consumption at room temperature [1,64]. Operando access to the domain correlation during or after voltage application is the key to the understanding of the involved dynamics and switching mechanisms. The seminal work from Lahtinen et al. demonstrated that one-to-one coupling of ferroelectric–ferromagnetic domains in an artificial multiferroic system can be addressed optically in the micrometer range using a combination of birefringent contrast imaging and magneto-optical Kerr effect (MOKE) microscopy (Figure 10a,b) [70]. Motion of magnetic domain walls driven by an electric field was recently demonstrated in perpendicularly magnetized Cu/Ni multilayers grown on BTO single crystals. López González et al. have shown that ferroelectric and ferromagnetic domain walls move in unison upon the application of out-of-plane electric field pulses. Neither a magnetic field nor an electrical current is required for this domain-wall motion, the velocity of which is hence determined by the electric field strength. 

In thin-film artificial multiferroic heterostructures, the nanoscale ferroelectric domain architecture cannot be optically resolved. Understanding the dynamics of magnetoelectric poling is, however, crucial for any technological implementations. De Luca et al. [93] shed some light on the magnetoelectric coupling dynamics between BFO and CoFe by employing SHG and MFM techniques operando, see Figure 10c–e. Ferroelectric and ferromagnetic domain states were investigated upon consecutive voltage applications. It was shown that the coupling between layers, as well as the domain pattern transfer, needs to be activated by an electric field in the order of the ferroelectric coercive field of BFO. Furthermore, spatially resolved SHG imaging through the magnetic electrode indicated the persistence of one-to-one ferroelectric–ferromagnetic domain correlation after voltage application. In this system, Manipatruni and colleagues [146] also demonstrated room-temperature voltage control of exchange coupling (uniaxial anisotropy) in giant magnetoresistance (GMR) spin valves coupled to multiferroic BFO. The BFO multiferroic imprint is absent in the pristine state, confirming a “wake-up” effect at the first electrical pulse [146]. Once activated, the device exhibits magnetoelectric hysteresis loops in good agreement with that of the ferroelectric hysteresis. Unidirectional anisotropy was confirmed by reversing the direction of the magnetoelectric effect upon sample rotation by 90°. 

## 5. Conclusions and Perspective

The experimental access to ferroic domain states is in ongoing progress. New concepts such as dynamical multiferroicity, i.e., generation of magnetization from varying electric polarization, could enable the non-invasive probe of domain wall movement using NV center magnetometry [147,148]. The development of non-invasive techniques for probing ferroelectric state in the ultrathin regime, e.g., synchrotron x-ray diffraction and optical second harmonic generation, pushes the establishment of new facets in the design of ferroelectric and multiferroic heterostructures. 

Furthermore, the emergence of polarization and related electrostatic effects can be visualized during deposition [35,149,150,151,152,153,154] because these materials may be grown epitaxially in the ferroelectric phase. Reflection high energy electron diffraction (RHEED) is the reference diagnostic tool for structural information during films synthesis but remains insensitive to the layer functionality. In situ SHG experiments directly access the ferroelectric polarization during thin film deposition of ferroic oxide multilayers [152]. Real-time, in-situ determination of the polarization state using SHG or x-ray diffraction in complex multilayer architectures [153] opens avenues towards the control of domain states in superlattices and the understanding to dynamics involved during the epitaxial design of ferroelectric multilayers. 

The domain visualization during the film deposition remains, however, a challenge. Improving spatial resolution in non-invasive probes would bring an understanding of multiferroic domain formation during the synthesis process. 

Moreover, recent works have shown that ferroelectric domain walls and multiferroic states can be deterministically tuned by optical means [155,156,157]. The demonstration of light-induced flexoelectric effect in multiferroic BFO thin films, i.e., driving a strain gradient with laser illumination, further reveals that light could be used to design new exotic polar states in oxide heterostructures, possibly during the growth process. The ability to probe and design new ferroic and multiferroic states during synthesis would enable new device paradigms relying on complex domain architectures. In situ control of ferroic switching events or domain nucleation can drastically accelerate the integration of complex oxide thin films into energy-efficient technologies.
